# Webina: an open-source library and web app that runs AutoDock Vina entirely in the web browser

**DOI:** 10.1093/bioinformatics/btaa579

**Published:** 2020-06-19

**Authors:** Yuri Kochnev, Erich Hellemann, Kevin C Cassidy, Jacob D Durrant

**Affiliations:** Department of Biological Sciences, University of Pittsburgh, Pittsburgh, PA 15260, USA; Department of Biological Sciences, University of Pittsburgh, Pittsburgh, PA 15260, USA; Department of Biological Sciences, University of Pittsburgh, Pittsburgh, PA 15260, USA; Department of Biological Sciences, University of Pittsburgh, Pittsburgh, PA 15260, USA

## Abstract

**Motivation:**

Molecular docking is a computational technique for predicting how a small molecule might bind a macromolecular target. Among docking programs, AutoDock Vina is particularly popular. Like many docking programs, Vina requires users to download/install an executable file and to run that file from a command-line interface. Choosing proper configuration parameters and analyzing Vina output is also sometimes challenging. These issues are particularly problematic for students and novice researchers.

**Results:**

We created Webina, a new version of Vina, to address these challenges. Webina runs Vina entirely in a web browser, so users need only visit a Webina-enabled webpage. The docking calculations take place on the user’s own computer rather than a remote server.

**Availability and implementation:**

A working version of the open-source Webina app can be accessed free of charge from http://durrantlab.com/webina.

**Supplementary information:**

Supplementary data are available at *Bioinformatics* online.

## 1 Introduction

Molecular docking is a popular computer-aided drug discovery (CADD) technique for predicting non-covalent small-molecule/macromolecular binding. By accelerating lead identification, docking aims to streamline the early-stage drug-discovery process. A docking program first predicts the 3D geometries (‘poses’) with which virtual-library compounds might bind a given macromolecular target. Second, a scoring function evaluates the predicted geometries to estimate binding affinities (Trott and Olson, 2009). The top-scoring compounds are then recommended for experimental testing. The resulting hit rates are far from 100%, but they tend to be better than those obtained from more costly high-throughput experimental screens (Muegge and Mukherjee, 2016).

Among docking programs, AutoDock Vina is particularly popular (Trott and Olson, 2009). Vina is an open-source program written in C++ that runs on all major desktop operating systems. Its strengths include speed and relative ease of use. But like many CADD programs, Vina has some notable shortcomings. Users must download and install the program to use it on their own machines. Choosing proper configuration parameters and analyzing Vina output is also sometimes challenging. And absent third-party graphical user interface (GUI) wrappers (Dallakyan and Olson, 2015; Di Muzio *et al.*, 2017; Lill and Danielson, 2011; Sandeep *et al.*, 2011; Seeliger and de Groot, 2010), Vina is only accessible from a Unix- or DOS-like command-line interface. These limitations are particularly impactful in educational settings, where expecting students to download, install and use a command-line program is often impractical.

To address these challenges, we created Webina, a WebAssembly (Wasm) library that runs AutoDock Vina entirely in a web browser. Wasm is an emerging web technology that allows developers to run compiled code without requiring the installation of any third-party plugins or programs. It can thus transform a stand-alone desktop computer program into a browser-compatible library that can be accessed via standard web applications. And because Wasm code runs CPU- and/or memory-intensive operations on the user’s own computer, those who create such web applications do not need to maintain the computer infrastructure typically required to run complex calculations on remote servers.

To facilitate use, we also created the Webina web app, which allows users to easily (i) configure/run the Webina library and (ii) visualize Webina-docked poses in their browsers. We also provide a separate web app called PDBQTConvert that allows users to convert their receptor/ligand input files from many popular formats (e.g. PDB, SDF, etc.) to the Webina/Vina-compatible PDBQT format (Jiang and Jin, 2017). The Supplementary Data describes how we compiled Vina to Wasm and created the Webina/PDBQTConvert web apps. It also provides detailed instructions for use.

## 2 Benchmarks

To compare Webina and Vina output and run times, we created a benchmark set of five protein/ligand complexes (see Supplementary Data). We intentionally selected complexes whose ligands are clinically approved and whose proteins have diverse structures and functions (Table 1).

**Table 1.** Webina/Vina benchmarks

PDB	Protein	Ligand	Webina RMSD/score	Webina time	Vina RMSD/score	Vina time
1HWL (Istvan and Deisenhofer, 2001)	HMG-CoA reductase	Rosuvastatin	0.8/-8.1	63.0	0.8/-8.1	22.3
2P16 (Pinto *et al.*, 2007)	factor Xa	Apixaban	1.3/-10.1	18.6	1.3/-10.1	14.3
3LN1 (Wang *et al.*, 2010)	COX-2	Celecoxib	0.6/-12.0	14.7	0.6/-12.0	8.9
4LL3 (Kozisek *et al.*, 2014)	HIV protease	Darunavir	2.3/-9.8	60.2	2.3/-9.9	45.0
4TZ4	cereblon	S-lenalidomide	2.3/-6.5	6.4	2.3/-6.5	4.0

*Note*: For both Webina and Vina, we considered only the top-scoring pose. RMSD refers to the root-mean deviation from the crytstallographic pose (in Å), calculated using the Open Babel *obrms* program. Scores are reported in kcal/mol. Times are reported in seconds.

### 2.1 Poses and scores

Webina and Vina 1.1.2 (macOS, 64-bit) produced nearly identical ligand poses and docking scores, as expected given that both are compiled from the same codebase and that we used the same random seed in all cases. For four of the five test complexes, the top-pose Webina and Vina docking scores were identical. For the fifth complex (HIV protease bound to darunavir), the scores differed by only 0.1 kcal/mol (Table 1). Webina and Vina also produced similar docked poses. In all five test cases, the top Webina and Vina pose differed by at most 0.21 Å (average 0.07 Å).

The slight differences in the Webina/Vina output are expected given that the two projects were created using different compilers. Indeed, the official pre-complied Vina binaries give slightly different outputs when run on Linux versus macOS. Regardless, we expect that the choice of random seed will have a far greater impact on docking than any differences resulting from the complication process.

### 2.2 Execution speed

Webina took longer to finish than did Vina 1.1.2, as expected given that it is a Wasm-compiled program. We tested Webina and Vina 1.1.2 (64-bit) on a MacBook Pro (15-inch, 2018) running macOS Mojave 10.14.5 (2.9 GHz Intel Core i9 processor and 32 GB 2400 MHz DDR4 memory). On average, Webina took roughly 1.75 times longer to dock the compounds than did Vina. When running a large-scale virtual screen, we recommend using the faster and more scalable command-line version of Vina. But when docking only a few compounds, Webina is an ideal, user-friendly solution that requires no installation or command-line use.

## 3 Conclusion

The Webina library aims to address usability challenges by running Vina entirely within the web browser. Our associated Webina web app, which leverages the Webina library, also provides user-friendly tools for setting up docking calculations (e.g. identifying an appropriate docking box) and analyzing docking output (e.g. examining predicted binding poses). Aside from allowing users to dock compounds in their browsers, Webina can also be used to examine the output of previously executed docking runs produced by Webina itself or command-line Vina. As further evidence of utility, the Supplementary Data provides two additional examples of Webina applied to medically relevant drug targets. It also compares our approach to the standard Vina executable, third-party GUIs, and docking server applications.

We have tested the Webina library on the browser/operating-system combinations shown in Table 2. Webina uses the SharedArrayBuffer JavaScript object to allow multiple processes/threads to exchange data directly. Most browsers disabled this object in 2018 due to concerns over the Spectre and Meltdown exploits. But it is currently available on Chromium-based browsers such as Google Chrome, and additional browsers (e.g. Firefox, Safari) are likely to re-enable SharedArrayBuffer soon.

**Table 2.** Browser compatibility

Browser	Operating system
Chrome 83.0.4103.14	macOS 10.14.5
Chrome 81.0.4044.113	Windows 10 Home 1903
Chromium 81.0.4044.92	Ubuntu 18.04.2 LTS

*Note*: We have tested Webina on Chromium-based browsers running on all major desktop operating systems.

Webina will be a useful tool for the CADD community. The Webina library and app are only 1.1 and 4.7 MB, respectively. We release both under the terms of the Apache License, Version 2.0. The independent PDBQTConvert app is only 4.4 MB and is released under the terms of the GNU General Public License, Version 2.0. Copies can be obtained free of charge from http://durrantlab.com/webina-download, and a public version of the web app is accessible at http://durrantlab.com/webina.

## Supplementary Material

btaa579_supplementary_data
