# Supplementary material for: Webina: an open-source library and web app that runs AutoDock Vina entirely in the web browser
Source: Bioinformatics. 2020 Jun 19;36(16):4513–5. doi: 10.1093/bioinformatics/btaa579 (PMC7575045; doi:10.1093/bioinformatics/btaa579)
Supplement: btaa579_supplementary_data [file btaa579_supplementary_data.pdf]

Supplementary Data

Webina: An Open-Source Library and Web App that Runs AutoDock Vina Entirely in the Web Browser

1 Webina Library: Compiling Vina to WebAssembly

To create the Webina library, we compiled the AutoDock Vina 1.1.2 codebase to WebAssembly (Wasm) using Emscripten 1.38.48, a toolchain specifically designed to port C/C++ applications to the web (https://emscripten.org). High-level languages such as C, C++, and Rust can be compiled to Wasm just as they can be compiled to operating-system-specific binary executable files. But Wasm-compiled programs run on a browser-based virtual machine rather than on a desktop operating system.

We compiled Vina to Wasm without modifying the original Vina 1.1.2 source code. Fortunately, Vina has only one dependency: the popular Boost libraries for C++. Many of these libraries are header-only, so including them in Emscripten-compiled projects requires no preliminary compilation. But Vina directly calls Boost libraries that are not header-only. These had to be first recompiled as static libraries for Wasm before compiling the main project.

The age of the Vina source code presented a major challenge. Vina has not been modified since 2011 and so is compatible only with older versions of Boost. The latest version of Boost is 1.71, but we used the *system, filesystem, serialization, program_options and thread/pthread* libraries from Boost 1.41. Aside from overcoming small bugs mostly related to C++11 incompatibilities, the key to successful compilation was to provide Emscripten with the required Boost include files. The resulting binaries had to be linked to *em++* during the compilation process by modifying the Vina Makefile. Further details can be found in the README.md file included with the Webina download.

Web-app developers can incorporate the library into their own computer-aided drug discovery (CADD) apps. The Webina git repository includes a simple example HTML file that demonstrates use. Vina command-line parameters are accessible via the Webina library, with the exception of the parameters used to specify input files. For security reasons, web browsers do not allow JavaScript to directly access users' file systems. Instead, the contents of local files must be read through a file input element, using a JavaScript FileReader object. To overcome this limitation, the Webina library accepts text strings containing the contents of the input PDBQT files, rather than the file paths normally specified via Vina parameters such as --receptor and --ligand.

2 Webina Web App

2.1 Typescript

To facilitate use, we have incorporated the Webina library into a convenient web app. The app is written in the open-source Microsoft TypeScript programming language. TypeScript is similar to JavaScript, but it includes additional features (e.g., optional variable typing, standard classes and interfaces, etc.) that are particularly useful when creating and maintaining complex codebases. TypeScript compiles to JavaScript and so can run in any modern web browser.

2.2 User Interface

The Webina web app leverages Vue.js 2.6.10 (https://vuejs.org/), an open-source JavaScript framework for building single-page web applications with consistent user interfaces. Vue.js allows web programmers to create their own reusable HTML-like components (e.g., text fields, buttons, etc.). The properties and values of each component may depend on user-initiated actions (e.g., a "Submit" button may only be enabled once the user has entered text into an associated form). With each user action, Vue.js automatically updates all dependent components throughout the entire app. The Vue.js framework thus greatly simplifies the process of creating complex web apps with many interconnected features.

We also use the open-source BootstrapVue library 2.0.2 (https://bootstrap-vue.js.org/) to ensure that all user-interface components have a consistent appearance. BootstrapVue provides a number of broadly useful Vue.js components (e.g., buttons, check boxes, pop-up message boxes, etc.). These components are styled according to the Bootstrap4 framework (https://getbootstrap.com/), which provides professionally designed templates that dictate components' color, size, and typography.

We also created a custom molecular-visualization Vue.js component that leverages the 3Dmol.js JavaScript library (Rego and Koes, 2015). This component allows the Webina web app to display macromolecular and small-molecule structures in the browser, as required for setting up Webina runs and analyzing Webina output. 3Dmol.js uses native web technologies to display these structures without requiring users to install any program or browser plugin.

2.3 Build Process

We use Webpack, an open-source module bundler, to organize and assemble custom and third-party libraries and files (https://webpack.js.org/). Webpack automatically builds the Webina web app by copying required files to their appropriate locations, combining files where possible, removing unnecessary/unused code, etc. Applying Google's Closure Compiler is a critical component of this build process (https://developers.google.com/closure/compiler). The Closure Compiler automatically analyzes and rewrites our TypeScript/JavaScript code to reduce file sizes and speed execution.

3 PDBQTConvert Web App

We created the PDBQTConvert web app to help novice users prepare their receptor/ligand files for Webina use. Like Vina, the Webina library accepts receptor/ligand input files only in the PDBQT format, a PDB-like format that additionally includes atom types and partial atomic charges. As described in the "Webina and Vina Benchmarks" section below, a number of command-line and other programs are available for converting receptor/ligand input files from popular formats (e.g., PDB, SDF, etc.) to PDBQT. But PDBQTConvert provides an easier way to prepare these files using a Bootstrap4-powered graphical user interface (GUI) in the browser. We distribute the PDBQTConvert app alongside Webina, but as an entirely separate program. Unlike the Webina library/app codebase, which is Apache-2.0 licensed, the PDBQTConvert app is GPLv2 licensed.

PDBQTConvert uses the OpenBabel JS library (Jiang and Jin, 2017) to convert receptor and ligand input files from many common formats (i.e., CAN, ENT, MCIF, MDL, MMCIF, MOL, MOL2, PDB, PQR, SD, SDF, SMI, SMILES, and XYZ) to the Webina-compatible PDBQT format. The app includes several user options. The first instructs PDBQTConvert to include branches and torsion trees, as required to indicate which ligand bonds should be considered rotatable during Webina/Vina docking. A second option instructs PDBQTConvert to add hydrogen atoms per a user-specified pH. Webina/Vina docking requires that receptor/ligand

PDBQT files include polar hydrogen atoms. But PDB receptor structures derived via X-ray crystallography often lack the resolution required to place hydrogen atoms, and many popular ligand formats (e.g., SMILES strings) do not enumerate explicit hydrogen atoms. A third option instructs PDBQTConvert to generate 3D coordinates for the input molecule. Many popular ligand formats (e.g., SMILES strings, flat SDF files, etc.) do not include 3D atomic coordinates, but Webina/Vina docking requires fully 3D models.

4 Webina Web App: Instructions for Use

4.1 Input Parameters Tab

To accommodate users who are not web developers, we have incorporated the Webina library into a Webina web app that includes additional tools for setting up Webina runs and visualizing docking results. On first visiting the Webina web app, the user encounters the “Input Parameters” tab. This tab includes several subsections that are useful for setting up a Webina run (Figure S1).

Input PDBQT Files. The “Input (PDBQT) Files” subsection allows the user to select their receptor and ligand files (Figure S1A). The user can also optionally specify a known-pose PDB or PDBQT ligand file. This file includes the ligand in its experimentally determined, correct bound pose (e.g., per X-ray crystallography or NMR). The known-pose file plays no role in the docking calculation; rather, it serves as a positive-control reference for evaluating Webina-predicted ligand poses. In our experience, it is often helpful to first benchmark Webina (or Vina) against a known ligand before using the program to evaluate compounds with unknown poses and binding affinities.

The “Input (PDBQT) Files” subsection also includes several options to simplify the process of preparing/testing protein/ligand input files. (1) If users wish only to test Webina without having to provide their own files, they can click the “Use Example Files” button to automatically load example receptor, ligand, and known-pose files. (2) If users specify receptor/ligand input files that are not in the required PDBQT format, the Webina web app will optionally attempt to convert them to PDBQT using the PDBQTConvert app. Interactions between the Webina and PDBQTConvert apps occur at “arm’s length” via an iframe. (3) If users’ receptor files include non-protein residues that might interfere with docking (e.g., a co-crystallized ligand), they can remove all non-protein atoms. (4) If users do not have a ligand file, they can use a web-based 2D molecular editor (Jiang *et al.*, 2016) to draw their ligand by hand. PDBQTConvert then converts that 2D ligand representation to a 3D PDBQT file for docking.

Docking Box. The “Docking Box” subsection allows users to specify the region of the receptor where Webina should attempt to pose the ligand (Figure S1B). This box-shaped volume is typically centered on a known pocket or cavity where a small-molecule ligand might reasonably bind.

To simplify the process of selecting a docking box, the Webina web app automatically displays 3D models of the user-specified receptor and ligand using the 3Dmol.js JavaScript library (Rego and Koes, 2015). By default, the receptor and ligand are displayed using cartoon and sticks representations, respectively. The user can toggle a surface representation as required to identify candidate receptor pockets. A transparent yellow box is superimposed on the structures to indicate the docking-box region.

When the user clicks the atoms of the receptor model, the Webina web app recenters the docking box on the selected atom. Users can also adjust the location and dimensions of the box using text fields below the molecular visualization.

Other Critical Parameters. The “Other Critical Parameters” subsection allows the user to specify the number of CPUs and the exhaustiveness setting (Figure S1C). We chose to set these two parameters

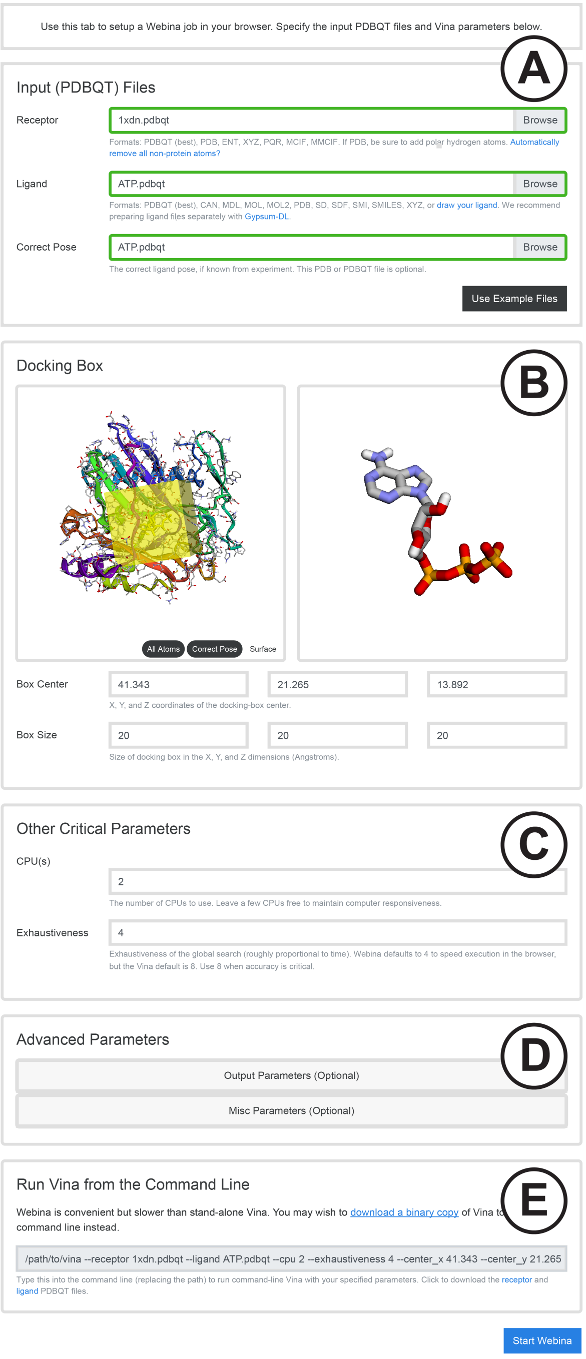

Fig. S1. The Input Parameters tab includes the A) Input PDBQT Files, B) Docking Box, C) Other Critical Parameters, D) Advanced Parameters, and E) Run Vina from Command Line subsections.

apart because they are particularly important in a browser-based setting. Users expect command-line tools to consume substantial computer resources, but they do not expect web apps to do so. By default, Vina uses all available CPUs and an exhaustiveness setting of eight. Webina has the same ability to consume CPUs and memory, but many users will wish to adjust these parameters to avoid impacting the performance of other programs and browser tabs.

Advanced Parameters. The “Advanced Parameters” subsection allows users to specify many additional parameters that are also available

via command-line Vina (Figure S1D). In our experience, it is rarely necessary to adjust these parameters, so they are hidden by default.

Run Vina from Command Line. The "Run Vina from Command Line" subsection aims to help Vina users who wish to use the Webina web app to setup their docking boxes and user parameters (Figure S1E). A text field provides a mock example of how to use command-line Vina with the specified parameters. Users can copy this example, modify it as needed, and paste it into their command-line terminals to run the desired calculation with the standard Vina executable. This subsection also includes links that allow the user to download the receptor/ligand PDBQT files for command-line use.

Starting the Webina Calculation. Once users click the "Start Webina" button, the Webina app will switch to the "Running Webina" tab while Webina executes. During this process, the user-specified receptor/ligand files and parameters are never transmitted to any third-party server. The calculations run entirely in the user's browser itself. The Webina browser tab may become unresponsive during execution as a result. Once the calculation is complete, the Webina web app will switch to the "Output" tab (described below) where users can visualize the docking results.

4.2 Existing Vina Output Tab

Beyond serving as a platform for running the Webina library, our Webina web app provides a useful interface for visualizing docking calculations. The "Existing Vina Output" tab allows users to load and visualize the results of previous Webina and Vina runs, without having to rerun the calculations. Users must specify the existing receptor and Webina/Vina output file they wish to visualize. They can also optionally specify a known-pose ligand file for reference. Users who wish to test the web app without providing their own files can click the "Use Example Files" button. Otherwise the "Load Files" button will open and visualize the specified files.

4.3 Output Tab

The "Output" tab allows users to visualize their Webina docking results (Figure S2). The same tab also displays the output of any previous Webina/Vina calculations that the user specifies via the "Existing Vina Output" tab.

Visualization. The "Visualization" subsection uses 3Dmol.js to display the receptor and docked molecule in cartoon/surface and sticks representation, respectively (Figure S2A). If the user has specified a known-pose ligand file, that pose is also displayed in yellow sticks. Like Vina, Webina predicts several poses per input ligand. A table below the visualization viewport lists each pose together with associated information such as the docking score. Clicking on a table row updates the 3D view with the specified pose so users can easily examine all predicted poses.

Output Files. The "Output Files" subsection shows the text contents of the Webina output files (Figure S2B). An associated "Download" button allows users to easily save those files.

Run Vina from Command Line. Similar to the "Input Parameters" tab, the "Output" tab also includes a "Run Vina from Command Line" subsection (Figure S2C). This subsection makes it easy for users to reproduce Webina's results using stand-alone Vina. It also reminds users what parameters they selected to generate the displayed Webina output.

4.4 Start Over Tab

The "Start Over" tab displays a simple button that allows the user to restart the Webina app. A warning message reminds the user that they will lose the results of the current Webina run unless they have saved their output files.

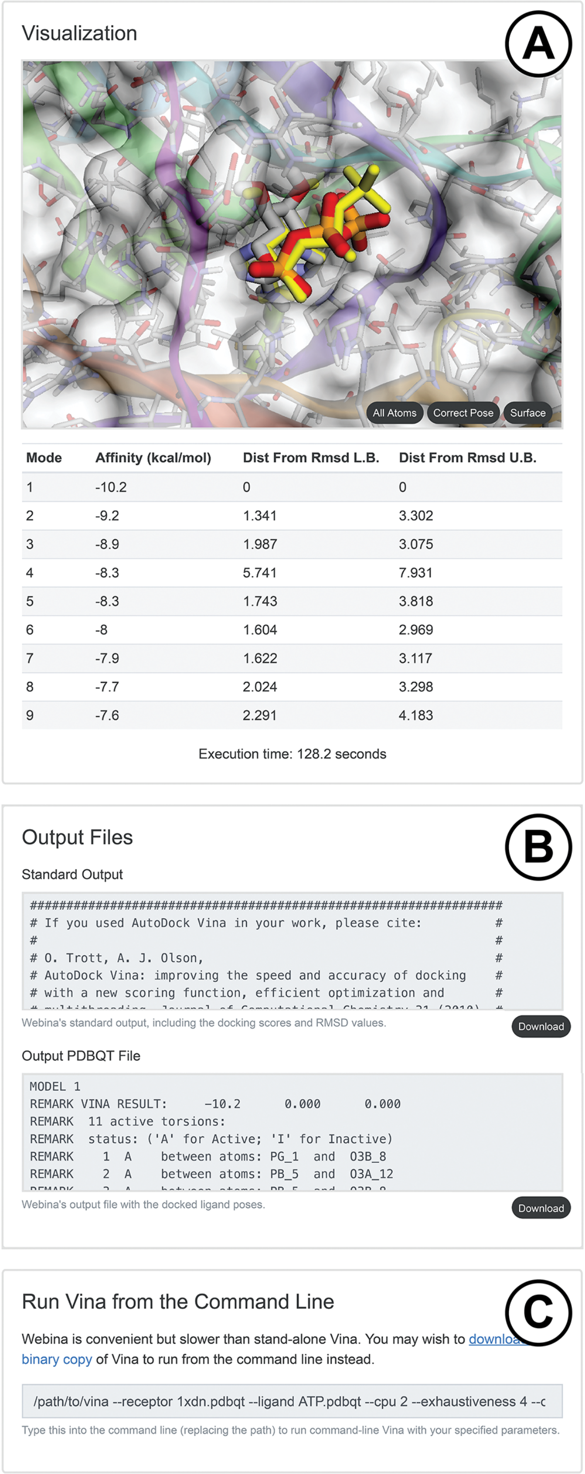

Fig. S2. The Output tab includes the A) Visualization, B) Output Files, and C) Run Vina from Command Line subsections.

5 Webina and Vina Benchmarks

5.1 Preparing Protein-Receptor Files

To compare Webina and Vina, we created a benchmark set of five protein/ligand complexes. To prepare the protein receptors for docking, we first removed all water molecules and ions. We then submitted the protein structures to the PDB2PQR server (Dolinsky *et al.*, 2004), a free resource

that adds hydrogen atoms per a user-defined pH while simultaneously optimizing the hydrogen-bond network. We used the default PDB2PQR settings, which assign protonation states at pH 7.

We next used Open Babel (O'Boyle *et al.*, 2011) to convert the output receptor PQR files back to PDB. Vina requires input receptor files to be in the PDBQT format. We used MGLTools 1.5.6 (http://mgltools.scripps.edu/), a desktop program created by the same group that created Vina, to convert the PDB files to PDBQT. We note that Open Babel can also convert files to the PDBQT format, but it does not always assign the same partial atomic charges and ligand rotatable bonds that MGLTools does.

One of the benchmark protein receptors, COX-2, contains heme groups. We similarly used MGLTools 1.5.6 to convert PDB files containing these heme groups to the PDBQT format. We appended these PDBQT-formatted files to the COX-2 PDBQT file prior to docking.

5.2 Preparing Ligand Files

We used the DrugBank database (Wishart *et al.*, 2006) to obtain the SMILES strings of each input ligand. We then used the open-source program Gypsum-DL (Ropp *et al.*, 2019) to generate protonated ligand models from these SMILES. For each input ligand, Gypsum-DL can generate multiple output PDB files that collectively account for alternate ionization, tautomeric, chiral, cis/trans isomeric, and ring-conformational states. To simplify our analysis, we instructed Gypsum-DL to generate only one molecular variant per input molecule, protonated as appropriate for pH 7. We again converted the Gypsum-DL output PDB files to the PDBQT format using MGLTools 1.5.6.

5.3 Webina and Vina Docking

We docked each ligand into its respective receptor using both Webina and Vina 1.1.2 (macOS 64-bit version). In each case, the docking box was centered on the geometric center of the corresponding crystallographic ligand, calculated using the *scoria* Python library (Ropp *et al.*, 2017). All docking boxes were 20 Å x 20 Å x 20 Å, cubed. Each docking run used four CPUs, an exhaustiveness setting of eight, and a consistent random seed of 123456789. The Webina git repository includes the receptor and ligand PDBQT files, for reproducibility's sake. We measured the root-mean-square deviation (RMSD) between each top-scoring docked pose and the corresponding crystallographic ligand using *obrms*, a program included in the Open Babel package (O'Boyle *et al.*, 2011).

6 Examples of Use

6.1 LARP1 Specificity

To demonstrate utility, we used Webina to study ligand binding to La-related protein 1 (LARP1), a potential anti-cancer drug target (Hopkins *et al.*, 2016; Mura *et al.*, 2015; Kato *et al.*, 2015). LARP1 regulates the translation of key proteins required for ribosome biogenesis (Fonseca *et al.*, 2018; Aoki *et al.*, 2013; Fonseca *et al.*, 2015; Philippe *et al.*, 2017; Tcherkezian *et al.*, 2014). The mRNAs that encode these proteins have a characteristic 5' terminal oligopyrimidine (TOP) motif, i.e., a cytosine invariably occupies the +1 position adjacent to the 7-methylguanosine (m^7G) cap, followed by a tract of pyrimidines (Meyuhas and Kahan, 2015). TOP mRNAs thus differ from most protein transcripts, which typically have a purine in the +1 position. The LARP1 DM15 region binds both the m^7G cap and the invariant +1 cytosine (+1C) characteristic of TOP motifs (Lahr *et al.*, 2015, 2017). We have previously shown using mutagenesis that the +1C pocket drives LARP1-TOP specificity (Cassidy *et al.*, 2019).

We used Webina to see whether we could predict LARP1 TOP-motif specificity *in silico*. We docked both the m^7GpppC and m^7GpppG dinucleotides into the 5V87:B DM15 structure, which was co-crystallized with bound m^7GpppC (Lahr *et al.*, 2017). The m^7GpppC and m^7GpppG ligands were prepared using Gypsum-DL as above, except the phosphate-bound hydrogen atoms were manually removed. The ligands were docked into a docking box with dimensions 16 Å x 17 Å x 17 Å, positioned so as to encompass both the m^7G- and +1C-binding pockets. To accommodate the flexibility of the triphosphate moiety, we ran Webina docking using an exhaustiveness setting of 20. Where two poses had the same predicted docking score, we favored the pose that placed the m^7G cap in the cap-binding pocket. See the Webina git repository for the input PDBQT files.

In harmony with previous experimental studies (Lahr *et al.*, 2017, 2015), Webina scored the top m^7GpppC pose slightly higher than the top m^7GpppG pose (-6.2 and -5.9 kcal/mol, respectively). Notably, the best m^7GpppC pose captures many of the same interactions seen crystallographically (Figure S3A) (Lahr *et al.*, 2017). The m^7G cap forms hydrogen bonds with E886, as well as π-π and cation-π interactions with Y922 and Y883. Similarly, the +1C forms hydrogen bonds with R847 and π-π stacking interactions with Y883 and F844. In contrast, the best m^7GpppG docked pose did not orient the +1G nucleotide such that its Watson-Crick face could interact with the +1C-pocket protein residues (Figure S3B).

We do not wish to overstate the utility of docking in general or Webina in particular. Docking certainly has its inaccuracies. For example, when we similarly docked the m^7GpppC and m^7GpppG dinucleotides into a LARP1 DM15 mutant engineered to specifically bind +1G transcripts (6PW3:C (Cassidy *et al.*, 2019)), the poses were not nearly as good (data not shown). But, typical docking inaccuracies aside, Webina is still a powerful, user friendly tool for generating biologically relevant hypotheses.

6.2 PARP-1 Ligand-Pose Prediction

As a second demonstration of utility, we used Webina to study Poly [ADP-ribose] polymerase 1 (PARP-1), a multifunctional nuclear protein involved in the base and nucleotide excision DNA repair pathways (BER and NER) (Bai, 2015). PARP-1 is overexpressed in various carcinomas and is associated with more aggressive cancer and lower survival rates (Malyuchenko *et al.*, 2015; Rojo *et al.*, 2011). BRCA-deficient carcinomas are especially susceptible to PARP-1 inhibition because they depend solely on BER for survival (Wang *et al.*, 2017).

To verify that Webina can effectively identify PARP-1 ligands, we first used it to recapitulate the binding poses of three crystallographically characterized PARP-1 inhibitors (positive controls): a benzo[*de*][1,7]naphthyridin-7(8*H*)-one derivative (PDB ID: 4HHY:A (Ye *et al.*, 2013)), niraparib (PDB ID: 4R6E:A (Thorsell *et al.*, 2017)), and rucaparib (PDB ID: 4RV6:A (Thorsell *et al.*, 2017)). We docked each ligand into all three crystal structures. All PARP-1 Webina runs used an exhaustiveness setting of 8. Docking boxes were centered on the PARP-1 catalytic domain. We used boxes with dimensions 12 Å x 20 Å x 14 Å, 15 Å x 20 Å x 14 Å, and 16 Å x 20 Å x 14 Å for the 4HHY:A, 4R6E:A, and 4RV6:A structures, respectively. See the Webina git repository for the input PDBQT files.

As expected, the best Webina-predicted binding affinity of each ligand was obtained when each was docked into its corresponding crystallographic conformation: the benzonaphthyridinone derivative, -14.7 kcal/mol; niraparib, -10.1 kcal/mol; and rucaparib, -10.8 kcal/mol. The heavy-atom RMSDs between the crystallographic and top-scoring docked poses were: the benzonaphthyridinone derivative, 2.35 Å; niraparib, 1.15 Å; and rucaparib, 0.77 Å.

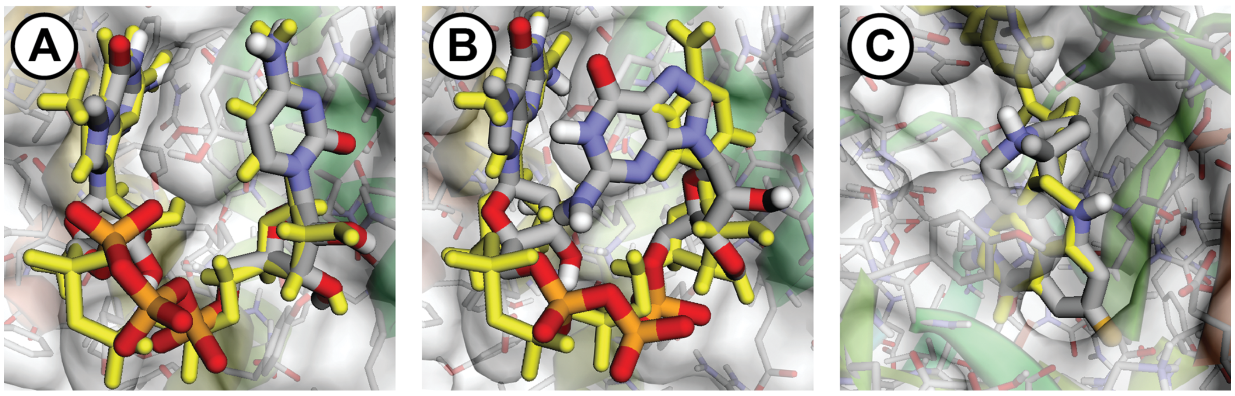

Fig. S3. Screenshots of Webina docking results. Docked poses are shown in sticks representation, colored by element. Crystallographic poses are shown in yellow sticks. A) The m^7GpppC/LARP1-DM15 docked pose recapitulates many of the hydrogen-bond, π-π-stacking, and cation-π interactions characteristic of the crystallographic pose. B) The m^7GpppG/LARP1-DM15 docked pose did not orient the +1G Watson-Crick face towards the protein. C) The pamiparib/PARP-1 docked pose is similar to the crystallographic pose of a benzonaphthyridinone ligand.

Having verified that Webina is effective in this context, we next used it to predict the binding pose of pamiparib, a PARP-1/2 inhibitor currently in phase II clinical trials (Friedlander *et al.*, 2019). We docked pamiparib into the same three PARP-1 conformations, using the same Webina parameters. The PARP-1 structure associated with the top-scoring pamiparib pose (-11.3 kcal/mol) was 4HHY:A (Figure S3C) (Ye *et al.*, 2013). In this docked pose, pamiparib shares much in common with the crystallographic poses of the three positive-control ligands. All four overlay a benzamide substructure at the same location (Figure S3C), enabling hydrogen bonds with S904 and G863 and a π-π stacking interaction with Y907. To the best of our knowledge, there is no publicly available PARP-1/pamiparib crystal structure. This work thus demonstrates how Webina can be used to prospectively predict ligand poses, a critical component of many CADD workflows.

7 Comparison with Other Approaches

7.1 Standard Vina Executable

Webina has several advantages over the standard Vina executable. Though code compiled to Wasm tends to run somewhat slower than natively compiled code, it runs entirely in the browser. And given that modern browsers already run on all major operating systems, Wasm-compiled programs are cross-platform by default. The Webina web app also provides a helpful GUI that command-line Vina lacks, making computer docking much easier for novices and students.

7.2 Third-Party Graphical User Interfaces

Several groups have created GUI-based desktop applications for setting up, performing, and analyzing Vina results. Many of these interact with command-line Vina "under the hood." For example, PyRx (Dallakyan and Olson, 2015), now a commercial program, provides a helpful wizard-like interface for Vina docking. Programs such as DockingApp (Di Muzio *et al.*, 2017) and AUDocker LE (Sandeep *et al.*, 2011) are somewhat similar (albeit less feature rich) open-source alternatives. Other GUI programs aim to simplify specific steps in the standard docking protocol. For example, MGLTools (http://mgltools.scripps.edu/), produced by the same group that created Vina, allows users to prepare ligand and receptor PDBQT files and to select an appropriate docking box. Popular molecular-visualization programs such as Chimera (Pettersen *et al.*, 2004) and PyMOL (DeLano, 2002) also include plugins (both commercial and free) for setting up and visualizing Vina docking runs (Lill and Danielson, 2011; Seeliger and de Groot, 2010).

These tools are certainly useful, but they still require users to download and install separate executable files. Furthermore, some of these tools are costly, work on only some operating systems, and still suffer from usability despite their GUI implementations. In contrast, Webina works entirely in the browser on all operating systems and is as easy to use as visiting a standard web page.

7.3 Server Applications

Recognizing the advantages of web-based docking, others have created server-based web apps that allow users to perform Vina docking on remote computer resources. MTiOpenScreen (Labbé *et al.*, 2015) is a good example of such an app. Though effective, the server-based approach requires app creators to maintain and secure the substantial remote resources needed to perform docking runs. Many users, especially those working in industry, may also be reluctant to upload their proprietary receptor and small-molecule structures to any third-party server. In contrast, Webina requires only one-way communication from the server to the browser. The browser downloads the Webina library just as it would an image or video. Webina then performs all docking calculations in the browser itself, without needing to transmit information about the docking input or output back to the server.

References

Aoki, K., Adachi, S., Homoto, M., Kusano, H., Koike, K., and Natsume, T. (2013). Larp1 specifically recognizes the 3' terminus of poly(a) mrna. *FEBS Letters*, **587**(14), 2173–8.

Bai, P. (2015). Biology of poly (adp-ribose) polymerases: the factotums of cell maintenance. *Molecular cell*, **58**(6), 947–958.

Cassidy, K. C., Lahr, R., Kaminsky, J. C., Mack, S., Fonseca, B. D., Das, S. R., Berman, A. J., and Durrant, J. D. (2019). Capturing the mechanism underlying top mrna binding to larp1. *Structure*.

Dallakyan, S. and Olson, A. J. (2015). *Small-Molecule Library Screening by Docking with PyRx*, pages 243–250. Springer New York, New York, NY.

DeLano, W. L. (2002). Pymol: An open-source molecular graphics tool. *CCP4 Newsletter On Protein Crystallography*, **40**, 82–92.

Di Muzio, E., Toti, D., and Polticelli, F. (2017). Dockingapp: a user friendly interface for facilitated docking simulations with autodock vina. *Journal of Computer-Aided Molecular Design*, **31**(2), 213–218.

Dolinsky, T. J., Nielsen, J. E., McCammon, J. A., and Baker, N. A. (2004). Pdb2pqr: an automated pipeline for the setup of poisson-boltzmann electrostatics calculations. *Nucleic Acids Research*, **32**(Web Server issue), W665–W667.

Fonseca, B. D., Zakaria, C., Jia, J. J., Graber, T. E., Svitkin, Y., Tahmasebi, S., Healy, D., Hoang, H. D., Jensen, J. M., Diao, I. T., Lussier, A., Dajadian, C., Padmanabhan, N., Wang, W., Matta-Camacho, E., Hearnden, J., Smith, E. M., Tsukumo, Y., Yanagiya, A., Morita, M., Petroulakis, E., Gonzalez, J. L., Hernandez, G., Alain, T., and Damgaard, C. K. (2015). La-related protein 1 (larp1) represses terminal oligopyrimidine (top) mrna translation downstream of mtor complex 1 (mtorc1). *Journal of Biological Chemistry*, **290**(26), 15996–6020.

Fonseca, B. D., Lahr, R. M., Damgaard, C. K., Alain, T., and Berman, A. J. (2018). Larp1 on top of ribosome production. *Wiley Interdiscip Rev RNA*, page e1480.

Friedlander, M., Meniawy, T., Markman, B., Mileshkin, L., Harnett, P., Millward, M., Lundy, J., Freimund, A., Norris, C., Mu, S., *et al.* (2019). Pamiparib in combination with tislelizumab in patients with advanced solid tumours: results from the dose-escalation stage of a multicentre, open-label, phase 1a/b trial. *The Lancet Oncology*, **20**(9), 1306–1315.

Hopkins, T. G., Mura, M., Al-Ashtal, H. A., Lahr, R. M., Abd-Latip, N., Sweeney, K., Lu, H. A., Weir, J., El-Bahrawy, M., Steel, J. H., Ghaem-Maghami, S., Aboagye, E. O., Berman, A. J., and Blagden, S. P. (2016). The rna-binding protein larp1 is a post-transcriptional regulator of survival and tumorigenesis in ovarian cancer. *Nucleic Acids Research*, **44**(3), 1227–1246.

Jiang, C. and Jin, X. (2017). Quick way to port existing c/c++ chemoinformatics toolkits to the web using emscripten. *Journal of chemical information and modeling*, **57**(10), 2407–2412.

Jiang, C., Jin, X., Dong, Y., and Chen, M. (2016). Kekule. js: an open source javascript chemoinformatics toolkit. *Journal of chemical information and modeling*, **56**(6), 1132–1138.

Kato, M., Goto, Y., Matsushita, R., Kurozumi, A., Fukumoto, I., Nishikawa, R., Sakamoto, S., Enokida, H., Nakagawa, M., Ichikawa, T., *et al.* (2015). Microrna-26a/b directly regulate la-related protein 1 and inhibit cancer cell invasion in prostate cancer. *International journal of oncology*, **47**(2), 710–718.

Labbé, C. M., Rey, J., Lagorce, D., Vavruša, M., Becot, J., Sperandio, O., Villoutreix, B. O., Tufféry, P., and Miteva, M. A. (2015). MTiOpenScreen: a web server for structure-based virtual screening. *Nucleic Acids Research*, **43**(W1), W448–W454.

Lahr, R. M., Mack, S. M., Heroux, A., Blagden, S. P., Bousquet-Antonelli, C., Deragon, J. M., and Berman, A. J. (2015). The la-related protein 1-specific domain repurposes heat-like repeats to directly bind a 5'top sequence. *Nucleic Acids Research*, **43**(16), 8077–88.

Lahr, R. M., Fonseca, B. D., Ciotti, G. E., Al-Ashtal, H. A., Jia, J. J., Niklaus, M. R., Blagden, S. P., Alain, T., and Berman, A. J. (2017). La-related protein 1 (larp1) binds the mrna cap, blocking eif4f assembly on top mrnas. *Elife*, **6**, e24146.

Lill, M. A. and Danielson, M. L. (2011). Computer-aided drug design platform using pymol. *Journal of computer-aided molecular design*, **25**(1), 13–19.

Malyuchenko, N., Kotova, E. Y., Kulaeva, O., Kirpichnikov, M., and Studitskiy, V. (2015). Parp1 inhibitors: antitumor drug design. *Acta Naturae*, **7**(3 (26)).

Meyuhas, O. and Kahan, T. (2015). The race to decipher the top secrets of top mrnas. *Biochim Biophys Acta*, **1849**(7), 801–11.

Mura, M., Hopkins, T. G., Michael, T., Abd-Latip, N., Weir, J., Aboagye, E., Mauri, F., Jameson, C., Sturge, J., Gabra, H., Bushell, M., Willis, A. E., Curry, E., and Blagden, S. P. (2015). Larp1 post-transcriptionally regulates mtor and contributes to cancer progression. *Oncogene*, **34**(39), 5025–36.

O'Boyle, N. M., Banck, M., James, C. A., Morley, C., Vandermeersch, T., and Hutchison, G. R. (2011). Open babel: An open chemical toolbox. *J. Cheminf.*, **3**, 33.

Pettersen, E. F., Goddard, T. D., Huang, C. C., Couch, G. S., Greenblatt, D. M., Meng, E. C., and Ferrin, T. E. (2004). Ucsf chimera - a visualization system for exploratory research and analysis. *Journal of Computational Chemistry*, **25**(13), 1605–1612.

Philippe, L., Vasseur, J.-J., Debart, F., and Thoreen, C. C. (2017). La-related protein 1 (larp1) repression of top mrna translation is mediated through its cap-binding domain and controlled by an adjacent regulatory region. *Nucleic Acids Research*, **46**(3), 1457–1469.

Rego, N. and Koes, D. (2015). 3dmol.js: molecular visualization with webgl. *Bioinformatics*, **31**(8), 1322–4.

Rojo, F., Garcia-Parra, J., Zazo, S., Tusquets, I., Ferrer-Lozano, J., Menendez, S., Eroles, P., Chamizo, C., Servitja, S., Ramirez-Merino, N., *et al.* (2011). Nuclear parp-1 protein overexpression is associated with poor overall survival in early breast cancer. *Annals of oncology*, **23**(5), 1156–1164.

Ropp, P., Friedman, A., and Durrant, J. D. (2017). Scoria: a python module for manipulating 3d molecular data. *Journal of Cheminformatics*, **9**(1), 52–58.

Ropp, P. J., Spiegel, J. O., Walker, J. L., Green, H., Morales, G. A., Milliken, K. A., Ringe, J. J., and Durrant, J. D. (2019). Gypsum-dl: an open-source program for preparing small-molecule libraries for structure-based virtual screening. *J Cheminform*, **11**(1), 34.

Sandeep, G., Nagasree, K. P., Hanisha, M., and Kumar, M. M. K. (2011). Audocker le: A gui for virtual screening with autodock vina. *BMC research notes*, **4**(1), 445.

Seeliger, D. and de Groot, B. L. (2010). Ligand docking and binding site analysis with pymol and autodock/vina. *Journal of computer-aided molecular design*, **24**(5), 417–422.

Tcherkezian, J., Cargnello, M., Romeo, Y., Huttlin, E. L., Lavoie, G., Gygi, S. P., and Roux, P. P. (2014). Proteomic analysis of cap-dependent translation identifies larp1 as a key regulator of 5'top mrna translation. *Genes & Development*, **28**(4), 357–71.

Thorsell, A. G., Ekblad, T., Karlberg, T., Löw, M., Pinto, A. F., Trésaugues, L., Moche, M., Cohen, M. S., and Schüler, H. (2017). Structural Basis for Potency and Promiscuity in Poly(ADP-ribose) Polymerase (PARP) and Tankyrase Inhibitors. *Journal of Medicinal Chemistry*.

Wang, L., Liang, C., Li, F., Guan, D., Wu, X., Fu, X., Lu, A., and Zhang, G. (2017). Parp1 in carcinomas and parp1 inhibitors as antineoplastic drugs. *International journal of molecular sciences*, **18**(10), 2111.

Wishart, D. S., Knox, C., Guo, A. C., Shrivastava, S., Hassanali, M., Stothard, P., Chang, Z., and Woolsey, J. (2006). Drugbank: a comprehensive resource for in silico drug discovery and exploration. *Nucleic Acids Res*, **34**(Database issue), D668–72.

Ye, N., Chen, C.-H., Chen, T., Song, Z., He, J.-X., Huan, X.-J., Song, S.-S., Liu, Q., Chen, Y., Ding, J., *et al.* (2013). Design, synthesis, and biological evaluation of a series of benzo [de][1, 7] naphthyridin-7 (8 h)-ones bearing a functionalized longer chain appendage as novel parp1 inhibitors. *Journal of medicinal chemistry*, **56**(7), 2885–2903.
